# Abscission checkpoint control: stuck in the middle with Aurora B

**DOI:** 10.1098/rsob.120095

**Published:** 2012-07

**Authors:** Mar Carmena

**Affiliations:** The Wellcome Trust Centre for Cell Biology, University of Edinburgh, Michael Swann Building, King's Buildings, Mayfield Road, Edinburgh EH9 3JR, UK

**Keywords:** Aurora B kinase, chromosomal passenger complex, endosomal sorting complex required for transport-III, cytokinesis, abscission, checkpoint

## Abstract

At the end of cell division, the cytoplasmic bridge joining the daughter cells is severed through a process that involves scission of the plasma membrane. The presence of chromatin bridges ‘stuck’ in the division plane is sensed by the chromosomal passenger complex (CPC) component Aurora B kinase, triggering a checkpoint that delays abscission until the chromatin bridges have been resolved. Recent work has started to shed some light on the molecular mechanism by which the CPC controls the timing of abscission.

## 2. Commentary

Unequal distribution of the genetic material during cell division can have serious consequences for the organism. Errors in chromosome segregation can lead to the formation of daughter cells containing missing or extra chromosomes. This condition—called aneuploidy—can have deleterious effects on cells and contributes to the process of carcinogenesis (reviewed in [1]). Even after successfully overcoming all the hurdles of nuclear division, the cell has to physically separate into two daughter cells through cytokinesis. Failure in cytokinesis results in tetraploid cells (with double the amount of chromosomes and centrosomes). Tetraploidy increases the frequency of chromosomal alterations and promotes tumour development in p53-null cells [2]. In non-transformed cells, tetraploidy normally triggers a G1 checkpoint mediated by the activation of the p53 pathway [3]. Those tetraploid cells that proceed to the next mitotic division sometimes manage to establish a bipolar spindle through centrosome clustering but otherwise are faced with the challenge of a multipolar mitosis. More often than not, this leads to aneuploidy. Cells have in place a series of surveillance mechanisms to ensure that the events of cell division are carefully orchestrated in order to avoid aneuploidy.

The chromosomal passenger complex (CPC) is one of the key regulators of cell division involved in the coordination of chromosomal and cytoskeletal events (for a recent review, see [4]). The enzymatically active member of the complex is Aurora B kinase, a member of the highly conserved Aurora family of Ser-Thr kinases [5]. INCENP (Inner Centromere Protein), Survivin and Borealin are the non-enzymatic subunits of the CPC, responsible for the targeting and full activation of the kinase. The CPC moves from the chromosome arms in early mitosis to the inner centromere by prometaphase–metaphase. At the metaphase–anaphase transition it translocates to the central spindle microtubules and later on is found at the midbody. This characteristically dynamic pattern of localization mirrors the movements of the kinase to different subcellular locations where it performs its multiple essential functions during mitosis and cytokinesis [4–6].

During the last decade, we have learned how the CPC contributes to the accuracy of mitosis by regulating kinetochore-microtubule attachments, the spindle checkpoint, central spindle assembly and chromosome compaction in anaphase [4,6]. We now also have a better understanding of the role of the CPC in the regulation of cytokinesis [7]. This last stage of cell division involves the formation of an actomyosin ring at the cell equator. The constriction of this ring drives the furrowing of the associated membrane, compressing the microtubules of the central spindle into the midbody and forming an intracellular bridge [8]. The CPC regulates multiple processes required for cytokinesis. Aurora B phosphorylation of centraspindlin components (MKLP1 and MgcRacGAP) is required for the formation and stabilization of the central spindle and for the activation of the small GTPase RhoA [4]. Aurora B also regulates cytoskeletal dynamics in cytokinesis (septins and intermediate filaments [9–11])

Aurora B is also involved in the regulation of abscission, the very last phase of cytokinesis. The final separation of the daughter cells involves the positioning of the abscission machinery, followed by the severing of the midbody microtubules and the final constriction and resolution of the plasma membrane [12]. Sometimes defects in chromosome architecture or DNA replication result in anaphase chromosome bridges that linger in the cleavage plane for longer than usual. Attempting cytokinesis in the presence of these bridges can lead to chromosome breakage and aneuploidy; sometimes cytokinesis fails, and the cell either becomes tetraploid or goes into apoptosis.

Cells have evolved a mechanism to avoid the progression of cytokinesis in the presence of a chromosome bridge, a checkpoint that delays abscission until the bridge has been resolved. The NoCut checkpoint was first described in *Saccharomyces cerevisiae*, where Ipl1/Aurora kinase localizes at the midzone and inhibits abscission through interaction with the Anillin-like proteins Boi1 and Boi2 [13]. In mammalian cells, Aurora B was also shown to be involved in an abscission checkpoint that avoids tetraploidization [14]. The downstream targets of Aurora B differ among organisms: it seems that some aspects of the molecular mechanisms underlying the control of abscission timing are not universally conserved. In addition, until recently, the link between Aurora B and the abscission machinery was unknown.

**Figure 1.**
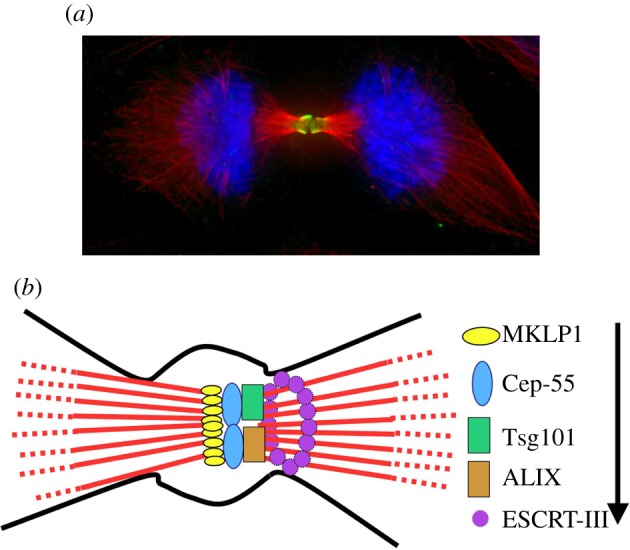
(*a*) Localization of Aurora B at the midbody in late mitosis in HeLa cells. Aurora B (green); tubulin (red); DNA (blue). (*b*) Diagram of the sequential recruitment of ESCRT complexes to the midbody. After PLK1 degradation dephosphorylated Cep-55 binds MKLP1. Cep-55 then will recruit Tsg101 (ESCRT-I) and ALIX, which in turn then recruit the ESCRT-III complex. ESCRT-III forms filaments around the abscission site that bring about the curvature of the membrane and drive the final cut.

Recent work has made a major contribution to our understanding of how Aurora B controls the timing of abscission through a direct interaction with the abscission machinery [15,16]. These reports showed that Aurora B acts through Shrb/CHMP4C, a subunit of the *e*ndosomal *s*orting *c*omplex *r*equired for *t*ransport (ESCRT-III) complex. The ESCRT complexes are proteins involved in membrane fission events. In humans, there are six complexes (ESCRT-0, -I, -II, -III, ALIX and VSP4). Their functions have been extensively described in multivesicular body formation and retroviral budding. Different ESCRT complexes are recruited sequentially to the site of scission, ending with the recruitment of the ESCRT-III complex that brings about membrane scission (figure 1*b*).

In cytokinesis, the ESCRT recruiter is Cep-55, a protein that interacts with the kinesin MKLP1 at the midbody. PLK1 phosphorylation negatively regulates the interaction of Cep-55 with MKLP1: only when PLK1 is degraded at the end of mitosis can Cep-55 interact with MKLP1 and accumulate in the midbody, allowing the initiation of abscission [17]. Cep-55 then interacts at the midbody with Tsg101 (ESCRT-I) and ALIX, which in turn recruit ESCRT-III, the complex responsible for the scission activity (figure 1*b*). ESCRT-III can assemble in filaments around the abscission site and make the membrane curve, eventually driving the final break between daughter cells.

In mammalian cells, Aurora B colocalizes at the midbody with CHMP4C, one of the subunits of ESCRT-III. CHMP4C is phosphorylated by Aurora B *in vivo*. It has been proposed that this phosphorylation drives localization of CHMP4C to the midbody where it can stop abscission [15]. Exactly how this happens is still subject to speculation. In their recent paper in *Open Biology*, Capalbo *et al.* point out that the Aurora B phosphorylation sites lie in a region that is required for the regulation of ESCRT-III activity. This would raise the interesting possibility that Aurora B phosphorylation could prevent a transition into an ‘open’ active form by inhibiting interaction with other regulators [16].

The paper by Capalbo *et al*. also introduces an interesting dimension to the role of the CPC in the control of Shrb/CHMP4C. Although in *Drosophila* the interaction between Shrb/CHMP4C and Borealin is conserved, the fly homologue Shrb neither contains the Aurora B phosphorylation sites nor is a substrate for this kinase. This prompts the authors to suggest an Aurora-independent role for Borealin in the regulation of abscission.

**Figure 2.**
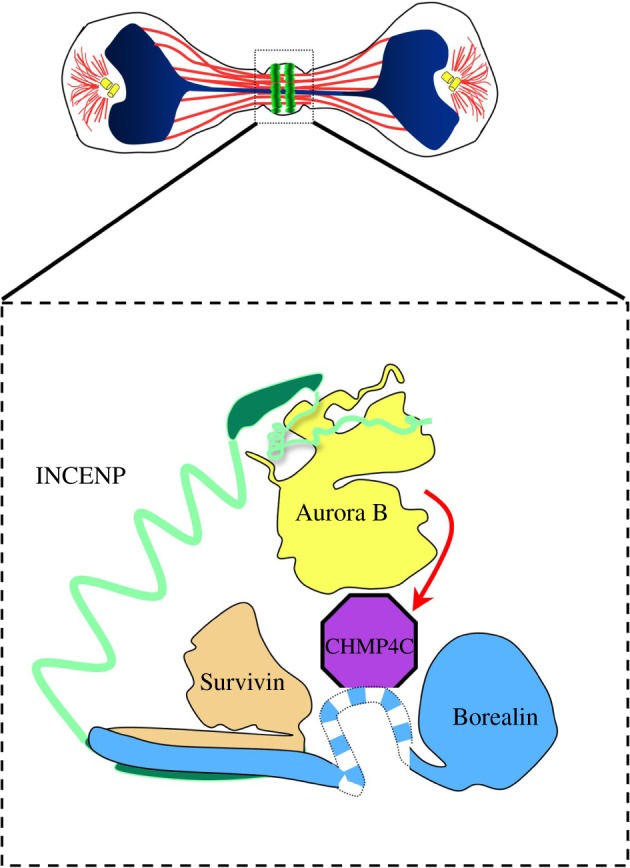
Dual role of Borealin in the control of ESCRT-III. In the presence of a chromosome bridge, the CPC delays the timing of abscission through regulation of ESCRT-III. Borealin may have evolved to play a dual role in this process: first, by bringing Aurora B into contact with its substrate CHMP4C (red arrow); second, by directly interacting with CHMP4C through its central domain (blue and white striped segment) and interrupting ESCRT-III filament formation.

Now how does this fit in with the present view of Borealin function? The N-terminus of Borealin forms a triple helical structure together with Survivin and the C-terminus of INCENP [18]. Borealin is thought to have a stabilizing role in this structure. The triple helical bundle is part of the localization module of the CPC, required for the targeting of the complex to its different locations in mitosis. Recently, NMR spectroscopy identified a dimerization domain at the C-terminus of Borealin. Phosphorylation of a residue at the interface of this domain by Mps1 kinase modulates dimerization and Aurora B activity [19,20]. The interaction of Borealin with CHMP4C occurs through the central region (amino acid 110–207) between these two functional domains (figure 2). We know that this unstructured central region could be exposed for interaction with other proteins because it contains sites that are phosphorylated by Cdk1 [21]. These phosphorylation events are required for the interaction of Borealin with the shugoshin proteins Sgo1 and Sgo2; this interaction is in turn required for the correct centromeric targeting of the CPC [21]. The findings of Capalbo and co-workers raise the interesting possibility that this region of Borealin performs an additional role in late mitosis. Therefore, in mammalian cells, Borealin could have a dual role: it would not only bring Aurora B in close proximity with its substrate CHMP4C, but also would interact directly with CHMP4C proteins and interfere with filament formation (figure 2). Future work will help us elucidate the exact mechanism by which Aurora B detects the chromatin bridges at the midbody and the extent of conservation of the roles of the CPC components in the regulation of abscission timing.
